# Northward Redistribution of Warm-Affinity Marine Fauna in the Iberian Atlantic: Insights from a Recent Northern Occurrence of the Hermit Crab *Dardanus calidus*

**DOI:** 10.3390/biology15191707

**Published:** 2026-09-24

**Authors:** Bruno Almón, Jose A. Cuesta, Héctor Costa, Rafael Bañón

**Affiliations:** 1 Centro Oceanográfico de Vigo, Instituto Español de Oceanografía (IEO-CSIC), Subida a Radio Faro, 50-52, 36390 Vigo, Spain; rafael.banon@ieo.csic.es; 2 Grupo de Estudo do Medio Mariño (GEMM), Puerto Deportivo s/n, 15960 Ribeira, A Coruña, Spain; 3 Instituto de Ciencias Marinas de Andalucía (ICMAN-CSIC), Avenida República Saharaui, 2, 11519 Puerto Real, Cádiz, Spain; jose.cuesta@csic.es; 4 IES Frei Martín Sarmiento, Av. Vigo 23 Pontevedra, 36003 Pontevedra, Spain; hecofer2@gmail.com

**Keywords:** marine biodiversity, biogeography, range shifts, Diogenidae, Galicia, tropicalization, barcoding

## Abstract

Poleward shifts in species distributions are increasingly reshaping marine communities worldwide. Along the Atlantic coast of the Iberian Peninsula, recent observations suggest ongoing biogeographical changes affecting a wide range of marine organisms. Here, we document the occurrence of the warm-water hermit crab *Dardanus calidus* in Galician waters (NW Spain), based on a single female collected in the region. Morphological and molecular analyses confirmed its identification, with the *COI* sequence showing high similarity to previously published Atlantic and Mediterranean sequences. The Galician record represents the northernmost occurrence of the species currently documented in the Atlantic Iberian region. Given the conspicuous nature of *D. calidus* and the comparatively well-documented hermit crab diversity of northwestern Iberia, the specimen is unlikely to reflect a common but previously overlooked component of the regional fauna. Although this single record does not demonstrate colonization or a sustained northward expansion, it adds to the increasing number of warm-affinity marine species recently reported beyond their historically documented ranges in northwestern Iberia and is consistent with broader evidence of ongoing biogeographical reorganization in the region. This finding highlights the value of integrating faunistic observations and molecular tools to track contemporary changes in marine species distributions.

## Simple Summary

The Atlantic coast of the Iberian Peninsula is currently experiencing important changes in marine biodiversity as species associated with warmer waters expand their distributions northwards. Here, we report the occurrence of the hermit crab *Dardanus calidus* in Galicia (northwestern Spain), approximately 460 km beyond the nearest area of the Portuguese coast from which the species has been regularly reported. Morphological and genetic analyses confirmed the identification of the specimen. Although this single record does not by itself demonstrate a sustained northward expansion of the species, it contributes to a growing body of observations indicating that marine species distributions in the region are becoming increasingly dynamic. Understanding whether these occurrences represent isolated dispersal events or persistent distributional changes is essential for predicting future responses of marine ecosystems to environmental changes and for supporting effective biodiversity conservation and management.

## 1. Introduction

Marine species distributions are undergoing rapid change worldwide, with poleward range shifts increasingly documented across multiple taxonomic groups and ocean basins [1]. These redistributions are among the most conspicuous biological responses to contemporary environmental change and are reshaping community composition, modifying species interactions, and altering historical biogeographical boundaries. Numerous marine taxa have expanded towards higher latitudes during recent decades, particularly in regions experiencing sustained ocean warming [2,3].

The Atlantic coast of the Iberian Peninsula is particularly well suited for detecting such changes. Located at the convergence of temperate, Lusitanian, Mediterranean and subtropical biogeographical influences, this region encompasses several distributional boundaries and transition zones for marine organisms [4]. Changes in community structure and species composition have been documented along the Portuguese coast, reflecting strong environmental gradients and the interaction of distinct biogeographical elements [5]. Oceanographic processes such as coastal upwelling, seasonal circulation and frontal dynamics further contribute to the complexity of this system and influence the connectivity of marine populations [6,7].

Particularly relevant is the central Portuguese coast, where the Cabo Espichel-Setúbal region has been associated with important shifts in species composition and distribution [4]. Rather than forming an absolute barrier, this sector appears to function as a zone of reduced connectivity in which coastal circulation patterns, upwelling intensity and larval transport processes influence the northward dispersal of many marine organisms [6,8,9]. Recent studies have increasingly recognized the importance of such oceanographically mediated transition zones in shaping marine biogeographical patterns and distributional limits [10].

Evidence accumulated during the last decades indicates that many historical distribution boundaries along the Iberian Atlantic are becoming increasingly dynamic [9,11,12]. Recent examples include the arrival of tropical and subtropical fishes in Galician waters [13], the appearance of several molluscan species beyond their historically documented ranges [14], and the northward expansion of warm-affinity macroalgae [4,15] and other marine organisms along the Portuguese and Galician coasts [16,17]. The growing number of species reported outside their previously known ranges supports the view that regional biodiversity is undergoing a process of biogeographical reorganization consistent with the ongoing tropicalization of marine communities reported elsewhere in the northeastern Atlantic [13,16].

The hermit crab *Dardanus calidus* (Risso, 1827) is a conspicuous warm-water member of the family Diogenidae, widely distributed throughout the Mediterranean Sea and the eastern Atlantic. Its Atlantic range extends from the Iberian Peninsula to the Cape Verde Islands and includes the Macaronesian archipelagos of the Azores, Madeira and the Canary Islands [18,19]. In the Atlantic Iberian region, published records are largely restricted to the southern and central Portuguese coast, with the Setúbal region representing the northernmost area from which the species has been repeatedly reported. Farther north, only a single historical record from Buarcos has been documented, based on material reported by Carvalho (1933, cited in Neves, 1977) [20]. Despite recent advances in the knowledge of hermit crab diversity and distribution in Spanish and Iberian waters, no previous confirmed record of the species from Galicia was identified in the published literature, regional inventories, taxonomic reviews, or biodiversity databases consulted. In this region, only four species of the family Diogenidae have previously been reported, with *Dardanus arrosor* (Herbst, 1796) as the sole congener known from the region [21,22]. Given its distinctive morphology, large size, and characteristic coloration, *D. calidus* represents a particularly suitable model for examining contemporary changes in species distributions and biogeographical patterns along the Atlantic coast of the Iberian Peninsula.

Here, we report the occurrence of *D. calidus* in Galician waters based on a specimen collected off Corrubedo (NW Spain) in July 2026. By combining morphological identification, molecular characterization and a biogeographical assessment of the record, we evaluate its significance within the broader context of ongoing northward redistribution of warm-affinity marine fauna in the northeastern Atlantic.

## 2. Materials and Methods

A single specimen of *Dardanus calidus* (Risso, 1827) was collected on 29 July 2026 off Corrubedo, Galicia, northwestern Spain (42°34.151′ N, 09°04.352′ W), at a depth of 6.5 m (Figure 1). The specimen was recovered as bycatch from a baited octopus trap deployed during routine artisanal fishing operations over a shallow coastal fishing ground characterized by a mixture of rocky and sandy substrate. The geographic coordinates were recorded using the fishing vessel’s GPS, and water depth was measured using its echo sounder. Trap deployment duration varies depending on fishing operations, although the traps are generally retrieved and checked at approximately 24 h intervals. No targeted survey was conducted following the capture, and no additional specimens have subsequently been reported to the authors.

After capture, the specimen was photographed fresh and preserved in 96% ethanol. A tissue sample from a pereopod was removed prior to preservation for subsequent molecular analyses. The specimen was deposited in the Marine Crustacean Collection (CRUST-IEOCD) belonging to the Spanish Institute of Oceanography (IEO-CSIC) [23], under catalogue number IEOCD-BR/3443.

Species identification was based on external morphological characters following available taxonomic descriptions and identification keys for eastern Atlantic and Mediterranean hermit crabs [18,24,25]. Diagnostic characters considered included overall body coloration, cephalothoracic armature, morphology of the ocular peduncles, relative size and ornamentation of the chelipeds, and the morphology of the ambulatory pereiopods.

The specimen was measured using digital calipers to the nearest 0.1 mm. Shield length (SL), measured from the tip of the rostrum to the posterior margin of the cephalothoracic shield, was used as the standard size metric.

Genomic DNA was extracted from muscle tissue at the Instituto de Ciencias Marinas de Andalucía (ICMAN-CSIC) using the Qiagen DNeasy Blood and Tissue Kit (Qiagen, Hilden, Germany) following the manufacturer’s protocol. A fragment of the mitochondrial cytochrome c oxidase subunit I (*COI*) gene was amplified. The primers used were LCO1490-JJ (5′-CHACWAAYCATAAAGATATYGG-3′) as forward and HCO2198-JJ (5′-AWACTTCVGGRTGVCCAAARAATCA-3′) as reverse primers [26]. PCR was conducted in 25 L volume reaction, containing 2.6 L (10×) of Surf buffer and 2.6 L Surf MgCl2 (25 mM), 1 L (20 M) of a mix of VWR dNTPs, 1 L (20 M) each of forward and reverse primer, 0.5 L (10 U/L) of Surt Hot Taq DNA polymerase (all Surf reagents from Stab Vida Laboratories, Portugal), 1 L of template DNA, and sterilized distilled water for molecular biology (PanReac ApplyChem, Castellar del Vallès, Spain) to a 25 L final volume. A negative control without template DNA was included in the amplification procedure. The amplification temperature profile consisted of an initial denaturation for 15 min at 95 °C, followed by 40 cycles of 45 s at 95 °C (denaturation), 45 s at 43 °C (primer annealing), and 45 s at 72 °C (elongation), and a final extension for 5 min at 72 °C. PCR products were sent to Stab-Vida Laboratories for purification and bidirectionally sequencing. Consensus sequences were inspected, assembled and edited using BioEdit v.7.0.5 [27]. Sequence quality was assessed by visual inspection of trace files using MEGA X v.10.2 [28], and ambiguous base calls and low-quality terminal regions were manually checked before generating the final consensus sequence. The final consensus sequence was translated into amino acids using the invertebrate mitochondrial genetic code to verify the absence of premature stop codons, frameshift mutations, and other obvious indications of sequencing errors or nuclear mitochondrial pseudogenes. Taxonomic validation involved comparing the resulting sequences against the NCBI BLASTn database [29] and also the Barcode of Life Data System (BOLD) Identification System [30]. The newly generated sequence has been deposited in GenBank under accession code PZ893448.

Distributional information for *Dardanus calidus* was compiled from published taxonomic and faunistic literature, regional inventories of the Galician and Iberian decapod fauna, and the review of Iberian hermit crabs by Almón et al. (2025) [22]. These sources were complemented by searches of GBIF, OBIS, and WoRMS, including the distributional sources linked to the species record. Records were checked against their locality, coordinates, date, identification, and original source whenever this information was available. Because individual occurrence points overlapped extensively in areas with a high concentration of records, the point layer was visualized using the Heatmap renderer available in the QGIS symbology settings. A radius of 7 mm was applied, with all records receiving equal weight. The resulting color gradient represents the relative spatial concentration of occurrence records, with darker red areas indicating greater concentration of records. This representation was used for visualization purposes only and should not be interpreted as a quantitative estimate of species abundance, occupancy, sampling effort, or habitat suitability. The distance between Corrubedo and the nearest repeatedly documented Portuguese locality (Setúbal) was calculated as a straight-line (Euclidean) distance using the QGIS distance measurement tool.

Digital artwork was prepared using Adobe Photoshop CS6, and the distribution map was generated using QGIS (version 3.44.9, https://qgis.org/).

## 3. Results

Family Diogenidae Ortmann, 1892
Genus *Dardanus* Paulson, 1875
*Dardanus calidus* (Risso, 1827) (Figure 2 and Figure 3)

**Material examined.** Spain, Galicia, Corrubedo • 1 ♀ SL 17.8 mm, adult non-ovigerous; 42°34.151′ N, 09°04.352′ W, 6.5 m depth, mixed rocky and sandy fishing ground; 29 July 2026 (IEOCD-BR/3443), recovered from a baited octopus trap. The specimen was collected alive from a shallow coastal fishing ground off Corrubedo (northwestern Spain). The individual occupied a shell of *Charonia lampas* (Linnaeus, 1758) approximately 12 cm in length, which hosted seven specimens of the symbiotic sea anemone *Calliactis parasitica* (Couch, 1842) (Figure 2a) covering the entire shell surface.

The record represents the northernmost occurrence of *Dardanus calidus* currently documented from the Atlantic Iberian region and extends the known Atlantic distribution of the species approximately 460 km beyond the nearest regularly reported localities on the Portuguese coast.

**Figure 2:**
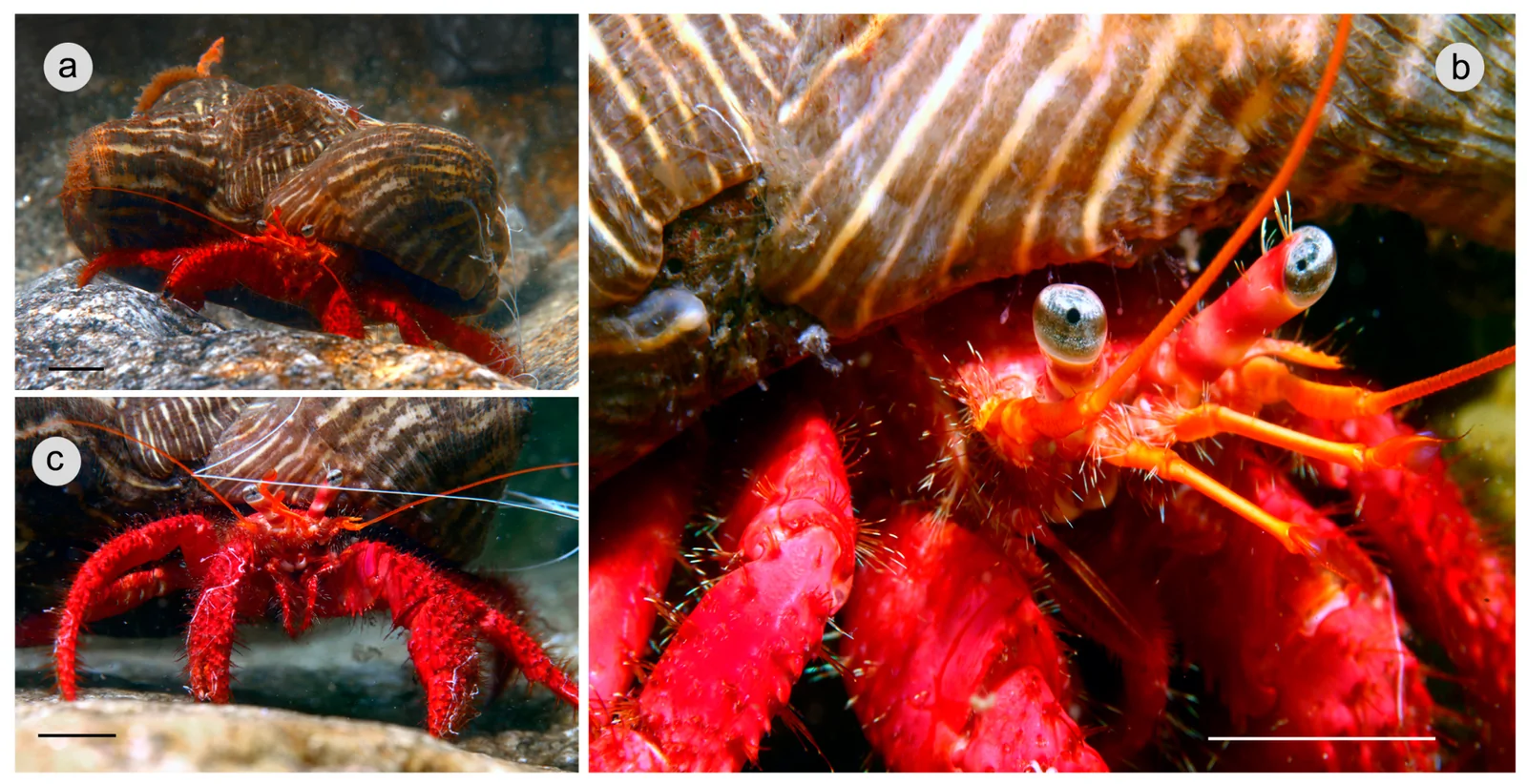
Morphological features of the Galician specimen of *Dardanus calidus*: (**a**) live individual occupying a shell of *Charonia lampas* associated with seven specimens of the sea anemone *Calliactis parasitica*; (**b**,**c**) general and detailed views of the same specimen showing the bright red coloration, dense setation and the unequal, robust and spinose chelipeds that characterize the species. Scale bars: (**a**–**c**) = 1 cm.

**Figure 3:**
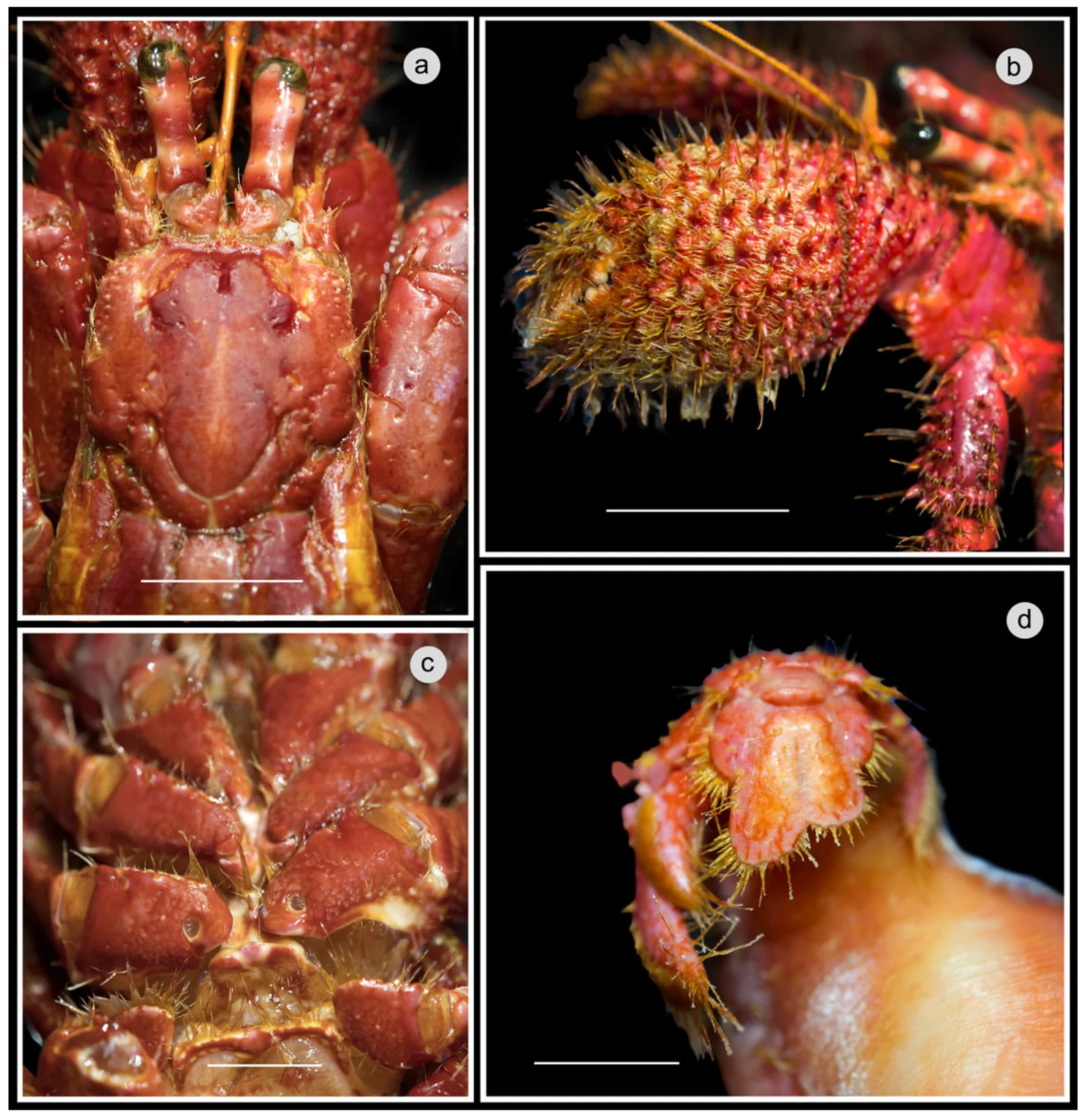
Morphological details of the Galician specimen of *Dardanus calidus*: (**a**) anterior part of the carapace and cephalic appendages, dorsal view; (**b**) left cheliped, outer view; (**c**) sternal area and coxae of the chelipeds and pereopods 2–4, showing the paired female gonopores; (**d**) telson, dorsal view. Scale bars: (**a**,**b**) = 1 cm; (**c**,**d**) = 5 mm.

### 3.1. Morphological Identification

The specimen was unequivocally identified as *Dardanus calidus* based on a combination of external morphological characters and live coloration (Figure 2 and Figure 3). Diagnostic features included the conspicuous bright red coloration of the body and appendages, the dense covering of yellowish setae on the chelipeds and ambulatory pereopods, and the markedly unequal chelipeds, with the left chela larger and more robust than the right (Figure 2b,c). The ambulatory pereopods bore conspicuous rows of strong spines and tubercles, whereas the ocular peduncles exhibited the characteristic color pattern described for the species, with four evenly spaced scarlet-red transverse rings on a pale yellowish background (Figure 2c and Figure 3a). The chelae were covered with regularly arranged robust spines with darkened tips (Figure 3b). The specimen was identified as an adult female based on the paired gonopores located on the coxae of pereopod 3 (Figure 3c); no attached eggs were observed. The telson was asymmetrical, with an acute median cleft on the posterior margin and five acute processes on each side, partially obscured by numerous long, stout setae (Figure 3d).

Together, these characters readily distinguish the species from *Dardanus arrosor*, the only congeneric species previously recorded from Galicia, and from all other hermit crab species recorded from Iberian waters. The two species differ in their overall live coloration, particularly in the color patterns of the ocular peduncles and appendages. However, the clearest distinction lies in the ornamentation of the chelipeds and ambulatory pereopods: those of *D. arrosor* bear distinctive parallel pectinate ridges with minute granules, rather than the robust, dark-tipped spines characteristic of *D. calidus*.

The association with a large gastropod shell colonized by multiple individuals of *Calliactis parasitica* is also consistent with the ecology commonly reported for the species (Figure 2a). Overall, the observed morphology agrees closely with published descriptions of *D. calidus* [18,24,25].

### 3.2. Molecular Characterization

A partial fragment of the mitochondrial cytochrome c oxidase subunit I (*COI*) gene was successfully obtained from the Galician specimen and deposited in GenBank under accession number PZ893448. Searches against GenBank and the Barcode of Life Data System (BOLD) unequivocally identified the specimen as *Dardanus calidus*. The sequence matched several Atlantic and Mediterranean specimens with identities ranging from 99.68% to 100% and was assigned to the BIN BOLD:AAE3682. The highest matches (100%) corresponded to specimens from the Azores and the Mediterranean Sea, while identities above 99.6% were also recovered for specimens from Turkey and additional Atlantic localities. The available comparative sequences ranged from 606 to 654 bp in length (see Supplementary Table S1), whereas the Galician sequence comprised 658 bp. Despite differences in total sequence length, the available sequences shared substantial homologous *COI* regions that allowed direct comparison, with query coverage ranging from 92% to 99% for the conspecific reference sequences.

The observed identities therefore confirm the close molecular affinity of the Galician specimen with previously reported Atlantic and Mediterranean specimens. However, the limited number and geographic representation of the available sequences preclude a detailed assessment of haplotype-level variation across the species’ range. In contrast, the closest non-conspecific sequence available (*Dardanus insignis*) showed only 87.69% sequence identity. These results confirm the molecular assignment of the Galician specimen to *D. calidus* and indicate no substantial divergence from previously reported Atlantic and Mediterranean sequences over the compared *COI* region.

Detailed BLAST (https://blast.ncbi.nlm.nih.gov/Blast.cgi) and BOLD (https://id.boldsystems.org/) identification results are provided in Supplementary Table S1.

## 4. Discussion

### 4.1. A Warm-Affinity Species Reaching Galicia

The occurrence of *Dardanus calidus* in Galician waters represents a substantial northward extension of the currently documented Atlantic distribution of the species and constitutes its first confirmed record from Galicia. Published Atlantic Iberian records of *D. calidus* are concentrated in southern and central Portugal, with only limited evidence of occurrences farther north reported by Carvalho (1933, cited in Neves, 1977) [20]. The presence of the species in Galicia is therefore biogeographically significant, regardless of whether it represents a recently established occurrence or a rare dispersal event.

The importance of this observation lies not only in the extension of the known distribution of *D. calidus*, but also in the geographical context in which it occurs. Galicia has increasingly emerged as a region where warm-affinity marine species are being recorded beyond their historically documented ranges across multiple taxonomic groups, including fishes, mollusks and macroalgae [13,14,15].

### 4.2. Historical Distribution Boundaries and Oceanographic Connectivity

Although the transport history of the Galician specimen cannot be reconstructed from the available data, the regional oceanographic setting provides relevant context for understanding the processes that may influence dispersal and historical distribution boundaries along the Atlantic Iberian coast.

The Atlantic coast of the Iberian Peninsula contains some of the most important biogeographical transition zones in Europe. In particular, central Portugal has long been recognized as an area where marked shifts occur in the composition of marine assemblages, reflecting the transition between temperate Atlantic communities and warmer Lusitanian and subtropical faunas [5].

Several studies have suggested that the Cabo Espichel-Setúbal sector plays a key role in shaping species distributions through its influence on larval transport and population connectivity. Coastal upwelling, frontal systems, offshore Ekman transport and coastline orientation interact to generate complex circulation patterns that can influence both larval retention and dispersal success [6,7]. More recently, empirical studies have demonstrated that these oceanographic processes can substantially affect larval delivery along the Portuguese coast [9], while theoretical models indicate that circulation-driven dispersal alone may be sufficient to generate persistent marine biogeographical boundaries [10].

However, such boundaries should not be viewed as absolute barriers. Instead, they are more appropriately interpreted as zones of reduced connectivity that limit, but do not entirely prevent, successful dispersal between adjacent regions [10]. Under this framework, the occurrence of *D. calidus* in Galicia suggests that dispersal beyond its historically documented Atlantic range remains possible. Although regional circulation may provide potential mechanisms for northward dispersal, the available data do not allow the Galician specimen to be linked to a particular transport pathway or oceanographic event.

Beyond central Portugal, the northwestern Iberian Peninsula represents an additional environmental transition characterized by strong seasonal upwelling, cooler average temperatures and distinctive hydrodynamic conditions. These conditions may influence the transport, arrival and persistence of warm-affinity species and may contribute to the maintenance of regional distributional boundaries. However, the relative importance of temperature, habitat availability, larval supply and circulation cannot be determined for *D. calidus* from the available data. Not all warm-affinity taxa that successfully reach northern Portugal subsequently establish populations in Galicia. Consequently, the occurrence of *D. calidus* in Galician waters is biogeographically relevant within the broader context of the increasing number of warm-affinity marine species recently documented in northwestern Iberia, although the process responsible for this particular occurrence remains unknown.

### 4.3. Recent Arrival or Previously Overlooked Occurrence?

An important question raised by this record concerns whether *D. calidus* represents a recent arrival to Galician waters or the detection of a previously overlooked occurrence. Although a rare or intermittent previous presence cannot be excluded, several arguments suggest that a recent arrival may be the more likely explanation.

Knowledge of Iberian hermit crab diversity has improved considerably in recent years through detailed taxonomic revisions and faunistic studies, and Galicia is among the regions for which distributional information is comparatively well developed. Indeed, the fauna of Iberian hermit crabs has recently undergone comprehensive taxonomic and faunistic revision [22], substantially reducing the likelihood that a conspicuous species such as *D. calidus* would remain undetected if regularly present in the region.

Furthermore, *D. calidus* is a particularly conspicuous species, characterized by its large size, intense red coloration and distinctive morphology. Given these characteristics, the lack of previous records despite decades of faunistic inventories suggests that the species is unlikely to have been a common component of Galician shallow-water assemblages. These considerations do not exclude an earlier rare occurrence, but make a regular and previously overlooked presence less likely.

Nevertheless, increasing observation effort should also be considered. Recreational diving, underwater photography, biodiversity reporting platforms and citizen-science initiatives have expanded substantially during the last two decades, increasing the probability that conspicuous species are detected and documented. Consequently, part of the apparent increase in records of warm-affinity marine organisms may reflect enhanced detectability rather than solely biological changes in species distributions [13,14].

### 4.4. Molecular Evidence and Genetic Affinity

The molecular analyses conducted in this study provide additional context for interpreting the Galician record, although their primary contribution is the independent confirmation of the specimen’s taxonomic identity. The newly generated *COI* sequence falls within the limited mitochondrial variation currently documented for *D. calidus* and does not constitute a markedly divergent *COI* lineage relative to the available Atlantic and Mediterranean sequences. Nevertheless, a single *COI* barcode cannot resolve population connectivity, geographic origin, or the timing of the Galician occurrence.

Among the mechanisms that could account for this record, larval dispersal appears biologically plausible given the planktonic development of marine decapods and the capacity of regional circulation to mediate transport over broad spatial scales [10,31]. The occurrence of *D. calidus* in Galicia could therefore result from larval transport from a previously documented occurrence area. However, the available evidence cannot identify the source or pathway of such dispersal, and a rare previously undetected occurrence cannot be excluded.

### 4.5. The Galician Record Within a Regional Biogeographical Pattern

The occurrence of *D. calidus* should ultimately be interpreted within the broader context of recent biogeographical change along the Atlantic coast of the Iberian Peninsula. Over recent decades, numerous warm-affinity species have been documented beyond their historically recognized ranges, contributing to changes in community composition and to the progressive tropicalization of regional marine ecosystems [1,2,3].

This phenomenon has become increasingly evident in northwestern Iberia, where recent studies have documented a growing number of fishes, mollusks and other marine organisms occurring beyond their previously reported ranges and, in some cases, reaching their northernmost Atlantic localities [13,14,17]. The occurrence of *D. calidus* in Galicia provides an additional example consistent with this broader regional pattern and extends it to a conspicuous benthic decapod whose occurrence had not previously been confirmed in the region.

The significance of the present record therefore lies not in independently demonstrating a sustained northward expansion, but in adding a morphologically and molecularly verified occurrence to an accumulating body of regional evidence. The record is consistent with the increasing dynamism of marine species distributions in northwestern Iberia, although the processes responsible for the occurrence of this particular individual cannot be determined from the available data. It may represent an isolated dispersal event, a rare previously undetected occurrence, or an early stage of a more persistent northward expansion. Repeated observations and targeted monitoring will be required to determine whether *D. calidus* persists in Galician waters and whether its occurrence represents a transient event or a sustained change in its Atlantic distribution.

## 5. Conclusions

The occurrence of *Dardanus calidus* in Galicia represents the northernmost record of the species currently documented in the Atlantic Iberian region, and adds a new example to the growing number of warm-affinity marine taxa reported beyond their historically recognized ranges in the Iberian Atlantic. Although based on a single individual, the record is noteworthy because it involves a conspicuous species whose occurrence in the region had remained undocumented despite considerable faunistic attention. Morphological and molecular evidence confirm its identification, and the *COI* sequence falls within the limited mitochondrial variation currently documented for the species.

This single occurrence does not independently demonstrate colonization or sustained northward expansion of *D. calidus*, and its origin and population status remain uncertain. Nevertheless, the record is consistent with the broader pattern of tropicalization and biogeographical reorganization supported by the increasing number of comparable observations across multiple marine taxa in northwestern Iberia.

As evidence accumulates across multiple taxonomic groups, northwestern Iberia is emerging as a sentinel region for understanding how marine species respond to changing environmental conditions. In this context, individual observations such as the one reported here provide valuable insights into the mechanisms and consequences of ongoing biogeographical change and reinforce the view that distributional boundaries in the northeastern Atlantic are becoming increasingly dynamic.

Continued monitoring of Galicia and adjacent Portuguese waters, including the documentation of repeated occurrences, juveniles or ovigerous females, will be essential to determine whether *D. calidus* represents an isolated occurrence, a previously undetected presence or the early stage of a sustained northward redistribution in the Atlantic Iberian region.

## Figures and Tables

**Figure 1:**
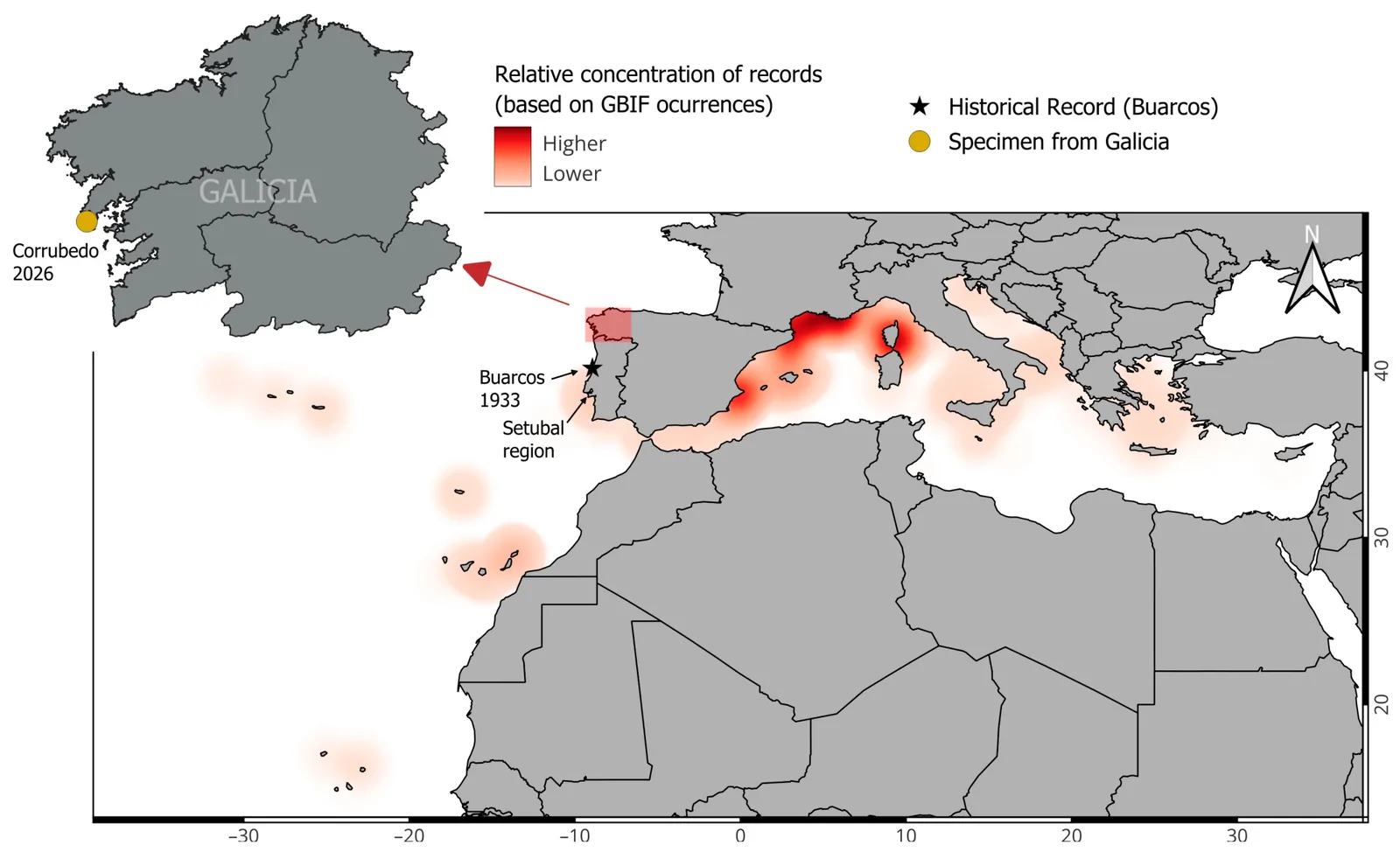
Distribution of *Dardanus calidus* in the eastern Atlantic and Mediterranean Sea based on GBIF occurrence records (https://doi.org/10.15468/dl.b8xh7u) and the published literature. The red color gradient represents the relative concentration of occurrence records, visualized using the QGIS Heatmap renderer with a radius of 7 mm. The heatmap scale ranges from lower to higher relative concentrations of occurrence records. The yellow dot indicates the new Galician record reported in this study. The black star corresponds to the isolated historical occurrence from Buarcos reported by Carvalho (1933, cited in Neves, 1977) [20].

## Data Availability

The original data presented in the study are openly available in GenBank under accession number PZ893448 and GBIF Occurrence Download https://doi.org/10.15468/dl.b8xh7u.

## Supplementary Materials

The following supporting information can be downloaded at: https://www.mdpi.com/article/10.3390/biology15191707/s1, Table S1. Closest matches obtained for the *Dardanus calidus COI* sequence generated in this study based on comparisons with sequences available in GenBank and in the Barcode of Life Data System (BOLD), including voucher code, geographic origin (location), accession numbers in GenBank and BOLD, sequence length (in number of bp), percent of sequence coverage, and percent of similarity.

## Abbreviation

GBIF	Global Biodiversity Information Facility
